# 4sc‐202 and Ink‐128 cooperate to reverse the epithelial to mesenchymal transition in OSCC

**DOI:** 10.1111/odi.13860

**Published:** 2021-05-04

**Authors:** Xi Yang, Tianyu Sun, Yajing Zhao, Shuying Liu, Xueyi Liang

**Affiliations:** ^1^ Department of Periodontics Stomatological Hospital Southern Medical University Guangzhou China

**Keywords:** epithelial‐mesenchymal transition, FoxO1, invasion and metastasis, oral squamous cell carcinom, Twist1

## Abstract

Treatment of oral squamous cell carcinoma remains a challenge due to a high incidence of treatment resistance, which is followed by tumor recrudescence and metastasis to the lymph nodes. Thus, it is important to explore novel inhibitors of OSCC. Here, we aimed to identify drugs that may cooperate with histone deacetylase inhibitors to reverse the EMT, inhibit EMT and cell migration and invasion, and contribute to therapeutic efficacy. We found that treatment with 4sc‐202 potently reversed the EMT and thereby inhibited cell migration and invasion in vitro, in part by inducing expression of the FoxO1 tumor‐suppressor gene. Furthermore, 4sc‐202 also synergized with Ink‐128 to inhibit tumor migration and invasion in vitro. Mechanistically, 4sc‐202 induced FoxO1 expression, whereas Ink‐128 promoted nuclear translocation of FoxO1. Our findings indicated that FoxO1 might reverse the EMT by interacting with Twist1 in OSCC. In conclusion, we identified an effective combination therapy involving class I histone deacetylase and mammalian target of rapamycin complex 1/2 inhibition that effectively blocked the EMT of tumor cells by upregulating FoxO1 expression to inhibit Twist1 transcription. These data have implications for developing new targets for early diagnosis and treatment of OSCC.

## 1 INTRODUCTION

Oral squamous cell carcinoma (OSCC) is the most common malignancy of the head and neck mucosa, and over 300,000 new cases are reported each year. Currently, the main treatment modalities for OSCC include surgical resection combined with radiochemotherapy (Siegel, 2018). Unfortunately, the 5‐year survival rates for patients with OSCC have remained less than 50% due to the high incidence of treatment resistance, with subsequent tumor recrudescence and metastasis to the lymph nodes (Ali et al., 2017). Therefore, the development of novel, effective therapeutic approaches for overcoming treatment resistance and metastasis has become a primary objective.

Gene expression profiling and other methods have identified gene expression changes related to the epithelial–mesenchymal transition (EMT) as significant factors in the development and/or progression of OSCC (Kurth et al., 2015). The EMT is a strictly regulated process during cancer progression, whereby epithelial cells lose their polarity and cell–cell adhesion, and acquire capabilities for migration and metastasis (Pastushenko et al., 2018). Many groups have confirmed that EMT promotes cancer cell migration and invasion, metastasis to the lymph nodes, and enrichment for a stem cell‐like cell phenotype (Pradella et al., 2017). Therefore, preventing the EMT is a key to curing OSCC and improving patient outcomes.

Post‐translational modification of chromatin histones is related to tumor progression and drug resistance in OSCC (Castilho et al., 2017). Histone deacetylases (HDACs) repress gene transcription by removing acetyl moieties from histones and, thus, they represent promising targets for anti‐cancer therapy (Malone et al., 2017). For OSCC, aberrant overexpression of class I HDACs is associated with poor prognosis (Chang et al., 2009), and inhibition of class I and II HDAC can effectively reduce invasion and metastasis (Liang et al., 2019). Consequently, HDAC inhibitors play inhibitory roles in cancer by reversing the EMT in many types of tumors (Fu et al., 2016; Pinkerneil et al., 2016; Zhijun et al., 2016). However, one of the potential limitations of single‐drug treatments in cancer is the development of chemotherapy resistance; the current view is that cancer treatments usually require combinations of targeted therapeutics to be effective in circumventing tumor resistance.

We therefore endeavored to identify other drugs that may cooperate with HDAC inhibitors to reverse the EMT, inhibit OSCC cell migration and invasion, and contribute to therapeutic efficacy. The drugs Ink‐128 (also known as mTORi) and 4sc‐202 (also known as HDACi) can effectively inhibit tumor growth. Here, we investigated the synergistic effect of combination treatment with both of these drugs in inducing FoxO1 and repressing Twist1 expression and thereby inhibiting EMT and tumor regression. The results of this study suggest a promising new combination therapy approach for OSCC and reveal a cooperative mechanism of action that could be more widely applied to develop other therapies.

## 2 MATERIALS AND METHODS

### 2.1 Cell lines, primary human tumor tissues, and compounds

The human OSCC cell line HSC6 was kindly provided by J. Silvio Gutkind (NIH, Bethesda, MD, USA). The resistant cell line CAL27R was kindly provided by Jingsong Li (Second Affiliated Hospital of Sun Yat‐sen University). Cell lines were identified by autosomal short‐tandem repeat profiling at either the ATCC or IDEXX Radil and were cultured in the recommended media. 4sc‐202 and Ink‐128 were purchased from Selleckchem.

### 2.2 Cell lysis and western blotting

Following appropriate culturing, proteins were extracted from whole cell lysates or cytoplasmic/nuclear lysate and analyzed by western blotting using polyclonal or monoclonal antibodies. All primary antibodies were used at 1:1,000–1:5,000 dilutions. The full scans of the western blots were visualized with a chemiluminescent and fluorescent imaging system. The relative quantities of protein were normalized to that of GAPDH, α‐tubulin, or H3. Information related to the antibodies used in this study is presented in Table S1.

### 2.3 Migration and invasion assay

Cell was resuspended in 500 µl of appropriate serum‐free medium at a concentration of approximately 1–2 × 10^5^ cells/ml and inoculated in the upper chambers of 24‐well plates that were precoated with or without 200 µl of BD Matrigel (250 µg/ml); 500 µl medium containing 10% fetal bovine serum was added to the lower chambers. After a 24‐hr incubation, the cells remaining in the upper inserts were removed gently using a cotton swab. The migrated and invading cells were fixed with 100% methanol for 10 min, stained with 0.1% crystal violet for 10 min, and quantified under a Zeiss microscope at 100× magnification in five randomly photographed fields.

### 2.4 Real‐time quantitative reverse transcription‐polymerase chain reaction (qRT‐PCR) analysis

Total RNA was extracted from cells in lysis buffer (Invitrogen) and reverse transcribed to complementary DNA using the Transcriptor First Strand cDNA Synthesis Kit (Roche), according to the manufacturer's instructions. qRT‐PCR was performed using SYBR Green I Master Mix (Roche) with a Light Cycler 480 system (Roche), as previously described(Wang et al., 2017). All results were normalized to GAPDH expression. Information regarding the specific primers used for qRT‐PCR is shown in Table S2.

### 2.5 Dual‐luciferase reporter assay

A luciferase reporter vector with a Twist1 promoter (Shanghai GenePharma) was used to test whether Twist1 was a direct target of FoxO1. The Twist1‐luciferase reporter, GP‐miRGLO, was constructed with either wild‐type or mutated FoxO1‐binding sites and transfected into cells. Luciferase activities were detected using the Dual‐Luciferase Reporter Assay System (Promega) with a Lumat LB 9507 luminometer (Promega), as previously described (Liang et al., 2019).

### 2.6 HDAC activity assay

The classⅠHDAC activity was measured by using Human Histonedeacetylase (HDAC) Elisa Kit according to the manufacturer's instructions.

### 2.7 Transfection with oeRNA or siRNA

Cells were transfected with the pGL3‐FOXO1 plasmid, the si‐FoxO1 plasmid, or a corresponding control plasmid using RNAiMAX (Invitrogen), according to the manufacturer's instructions. The medium was changed after transfection with the pGL3‐FOXO1 plasmid, the si‐FoxO1 plasmid, or a corresponding control plasmid for 16 hr. The RNA‐oligonucleotide and DNA‐decoy sequences were synthesized by Sangon Biotech.

### 2.8 ChIP assay

ChIP assays were performed using a ChIP Assay Kit (Millipore, USA), as described previously (Zhuang et al., 2017). Briefly, cells were cross‐linked with 1% formaldehyde (in phosphate‐buffered saline) for 10 min at room temperature, lysed in lysis buffer, and sonicated using a VCX130 device (SONICS) at a power level of 25% with 5‐s pulse at 9‐s intervals. Agarose gel electrophoresis revealed that the resulting DNA fragments were between 200 and 1,000 bp in length. The chromatin extract was immunoprecipitated overnight at 4°C with protein A/G‐agarose beads, using an antibody against Twist1 or an isotype‐matched control immunoglobulin G (IgG). For qPCR analysis, 2.5 µl of the immunoprecipitated DNA or 2% of the reference material was used as the template. The PCR results were normalized using the input DNA. The sequences of the primers used for ChIP‐qPCR are shown in Table S3.

### 2.9 RNA‐Sequencing analysis

RNA fragments of 50 nucleotides (subjected to paired‐end reading) were analyzed using a poly(A)‐based library (BGI, Shenzhen, China). Using the RSEM software package, the RNA levels were quantified in terms of the reads per kilobase of transcript, per million mapped reads. For genome analysis, we used TopHat 2.0.1 software to detect differential expression of the transcripts (Cuffdiff/Cufflink 2.2.0). Transcripts with a >2‐fold change in expression were analyzed using the Kyoto Encyclopedia of Genes and Genomes (KEGG) database to determine their functions or pathway associations. A heat map was generated using R software (https://www.r‐project.org/).

### 2.10 Statistical analysis

GraphPad Prism 5.0 software (La Jolla, CA, USA) was used for all statistical analysis. All data are presented as means ± standard deviations for at least triplicate samples, unless stated otherwise. Statistical significance was analyzed using Student's *t* test, or Tukey's test after ANOVA, as appropriate. A *p*‐value of <.05 was considered to reflect a statistically significant difference.

## 3 RESULTS

### 3.1 4sc‐202 blocked the invasion and migration capabilities of OSCC cells

**FIGURE 1:**
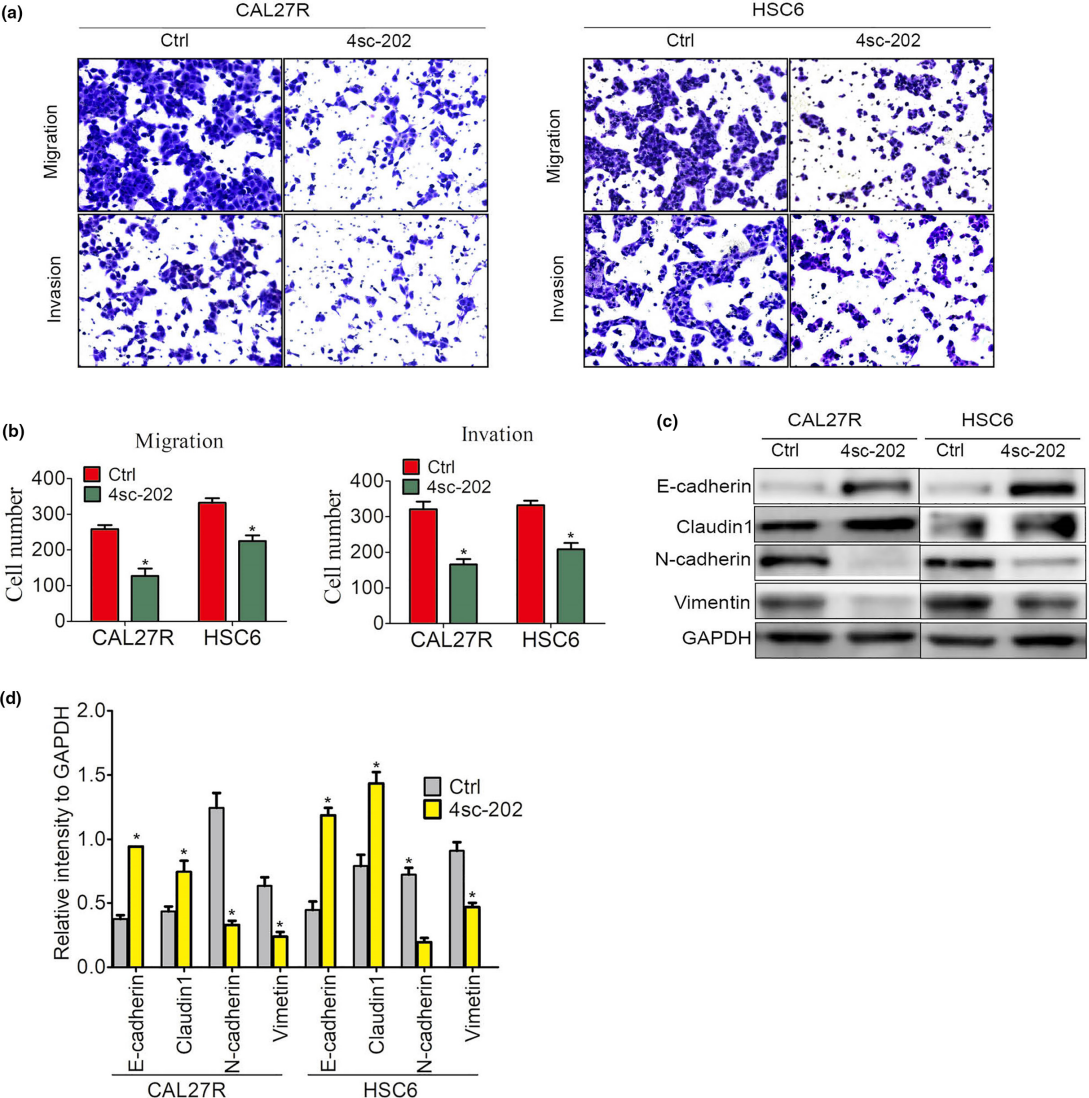
4sc‐202 inhibited OSCC cell invasion and migration in vitro. (a, b) CAL27R and HSC6 cells were cultured for 24 hr in the absence or presence of 4sc‐202 at a dose (1 µM) that was far below the 50% lethal inhibition concentration (IC_50_) (CAL27R IC_50_: 1.65 μM, HSC6 IC_50_: 2.29 μM), and the migratory and invasive abilities were examined by performing Transwell assays. Representative migrated or invaded pictures to the lower surface (a) and the mean number of migrated or invaded cells were shown (b). (c, d) EMT‐associated markers, E‐cadherin, claudin‐1, N‐cadherin, and vimentin were examined by western blotting after cells were treated with or without 1 μM 4sc‐202 for 24 hr (c), and the relative expression intensities compared to the GAPDH internal control are shown (d)

To investigate the therapeutic potential of 4sc‐202 against OSCC, we utilized CAL27R and HSC6 cell lines, which possess relatively high metastatic abilities. Our transwell assay results showed that 4sc‐202 inhibited the migration of CAL27R and HSC6 by 67.7% and 49.3%, respectively, and inhibited the invasion by these cells by 63.6% and 51.6%, respectively (Figure 1a,b). Similar effects were observed in other five OSCC cell lines: CAL33, SCC25, SCC15, SCC9, and UM1 (Figure S1a–f). In addition, 4sc‐202 showed increased expression of epithelial cell markers (e.g., E‐cadherin and claudin1) and decreased expression of stromal phenotype markers (e.g., N‐cadherin and vimentin) (Figure 1c,d). Therefore, these results indicate that 4sc‐202 blocked OSCC invasion and migration in vitro.

### 3.2 FoxO1 was induced by 4sc‐202 and could reverse OSCC EMT

**FIGURE 2:**
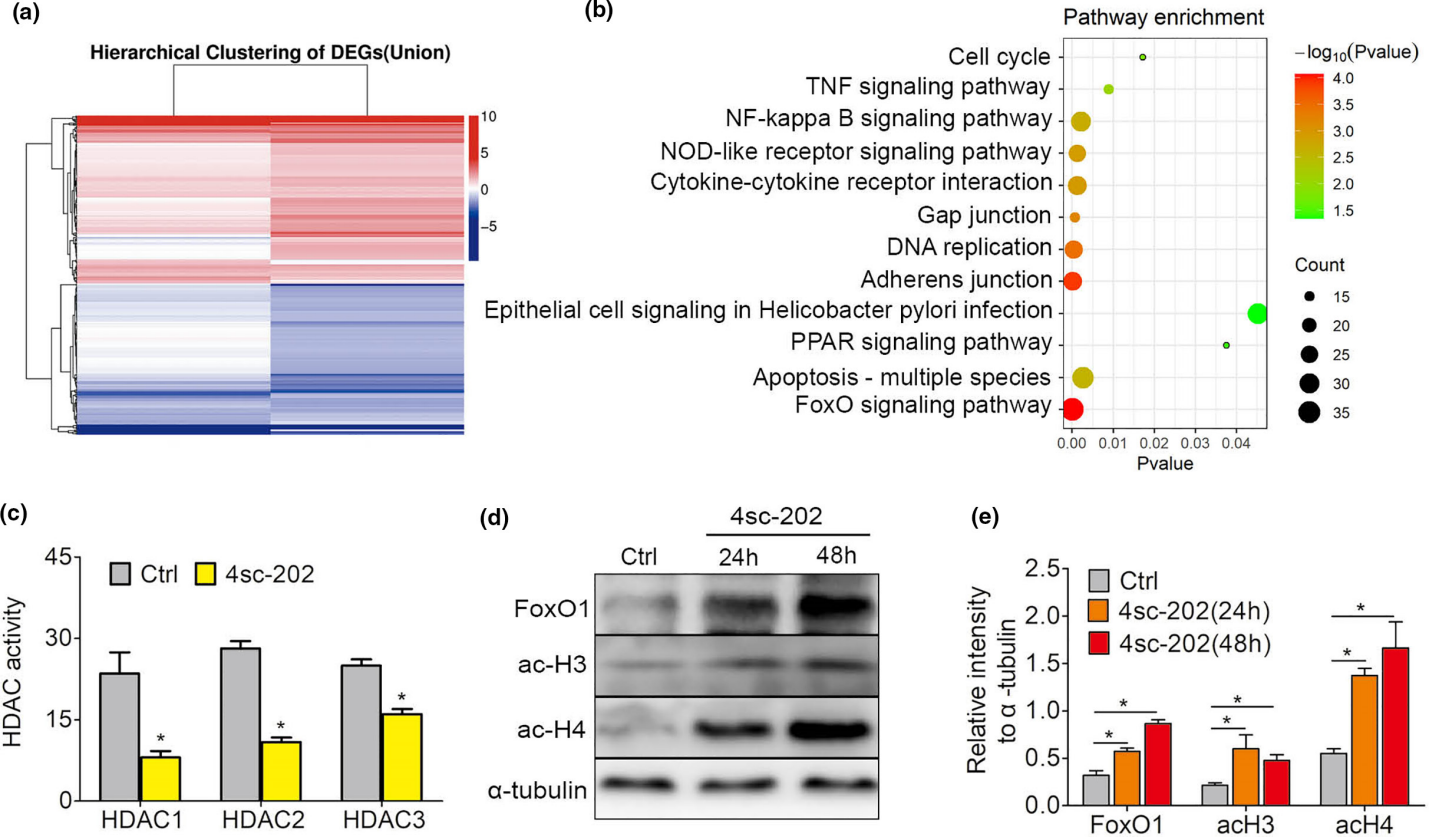
Expression profiling identifies FoxO1 as a target of 4sc‐202 in OSCC. (a) Unsupervised hierarchical clustering of genes differentially expressed between HSC6 cells treated with or without 1 μM 4sc‐202 for 24 hr. Each row represents an individual differentially expressed gene (fold change ≥1.5; *p* < .05). (b) KEGG analysis was performed to generate a list of enriched pathways affected by 4sc‐202 treatment in HSC6 cells. (c) The lower HDAC enzyme activity is observed upon 4SC‐202 treatment compared to control. (d, e) Expression levels of FoxO1, ac‐H3, and ac‐H4 in HSC6 cells transfected with si‐NC or si‐FoxO1 were detected by western blotting (d), and the relative expression intensities compared to the α‐tubulin internal control are shown (e)

To examine the molecular mechanism underlying the anti‐cancer capacity of 4sc‐202, we analyzed the transcriptome of HSC6 cells treated with 4sc‐202. We identified 611 upregulated and 2,423 downregulated genes with a >1.5‐fold increase after 4sc‐202 treatment (Figure 2a). KEGG analysis revealed significant enrichment for mRNAs involved in signaling networks, including the NF‐kappa B signaling pathway, the FoxO1 signaling pathway, and the TNF signaling pathway (Figure 2b). We observed dramatic upregulation in the expression of FoxO1, which coordinates EMT‐related pathways and EMT‐related transcription factors (EMT‐TFs), and thereby modulates the EMT process. Since 4sc‐202 belongs to class I HDAC inhibitors which upregulate target genes through promoting histone acetylation near the target gene transcription start site (Figure 2c), we speculated that 4sc‐202 activates FoxO1 via histone acetylation. Our research revealed that4sc‐202 treatment induced the levels of acetylated histones (H3 and H4) (Figure 2d,e).

**FIGURE 3:**
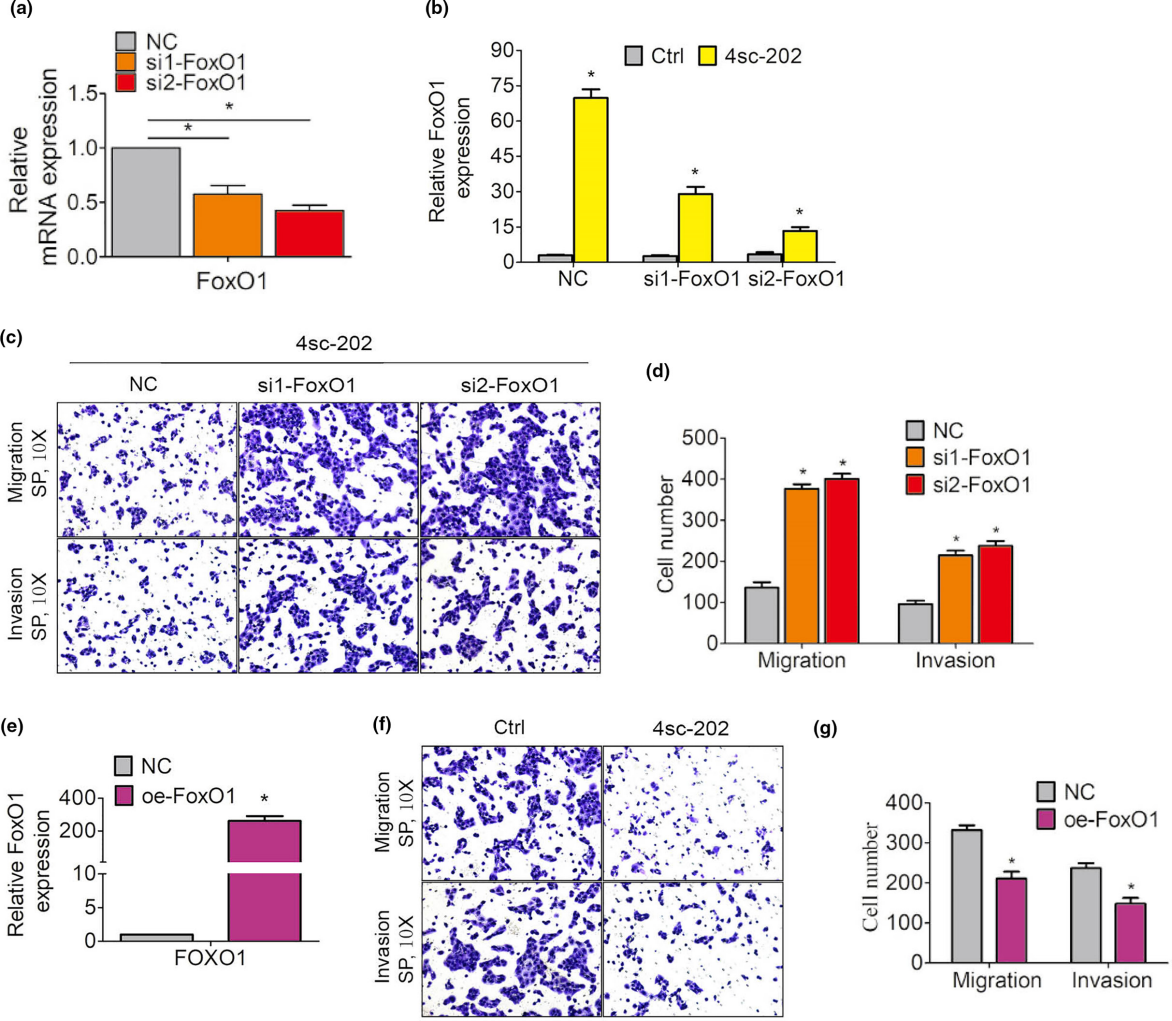
FoxO1 levels affected the efficacy of 4sc‐202 against OSCC EMT. (a–d) HSC6 cells transfected with si‐NC or si‐FoxO1. (a) mRNA expression levels of FoxO1 were measured by qRT‐PCR. (b) Cells were treated with DMSO or 4sc‐202 for 24 hr, and FoxO1 mRNA levels were measured by qRT‐PCR. (c, d) Cells are treated with the indicated concentrations of 4sc‐202 for 24 hr, and their migratory and invasive abilities were examined by performing transwell assays. Representative images showing migration or invasion to the lower surface (c) and the mean number of migrated or invaded cells were shown (d). (e–g) HSC6 cells transfected with an empty plasmid or a vector encoding oe‐FoxO1. (e) FoxO1 expression levels were measured by qRT‐PCR. (f) The migratory and invasive abilities were examined by performing transwell assays. Representative images showing migration or invasion to the lower surface (f) and the mean number of migrated or invaded cells were shown (g)

To test whether FoxO1 levels affect the efficacy of 4sc‐202 on OSCC EMT, we transfected OSCC cells with a negative control small‐interfering RNA (si‐NC) or si‐FoxO1 (Figure 3a). As shown in Figure 3b, 4sc‐202 induced FoxO1 expression in si‐NC transfectants, which was attenuated by knocking down FoxO1 expression. After 24 hr, si‐FoxO1 significantly reduced the sensitivity to 4sc‐202 in terms of the invasive and migratory capacities of OSCC, compared with si‐NC (Figure 3c,d). To test whether FoxO1 was sufficient for blocking OSCC EMT, we used a plasmid encoding the full‐length FoxO1 to transiently overexpress FoxO1 in OSCC cells (Figure 3e). After 24 hr, FoxO1‐expressing HSC6 cells showed a 63.5% decrease in migration and a 62.4% decrease in invasion, when compared to control cells (Figure 3f,g). These data indicate that FoxO1 induction promoted 4sc‐202‐mediated inhibition of OSCC EMT.

### 3.3 Co‐treatment with 4sc‐202 and Ink‐128 synergistically induced FoxO1 and blocked EMT in OSCC cells

FoxO1 phosphorylation promotes its translocation to the cytoplasm, which can be modulated by the AKT‐mTOR pathway (Ma et al., 2018). Previous research showed that an antagonist of this pathway, Ink‐128, inhibited OSCC metastasis (Wang et al., 2017). The finding that treatment with 4sc‐202 and Ink‐128 both restrained OSCC EMT and increased FoxO1 activity (4sc‐202 promoted FoxO1 expression and Ink‐128 accelerated nuclear FoxO1 translocation) suggests that FoxO1 is a crucial determinant of OSCC EMT regulation.

**FIGURE 4:**
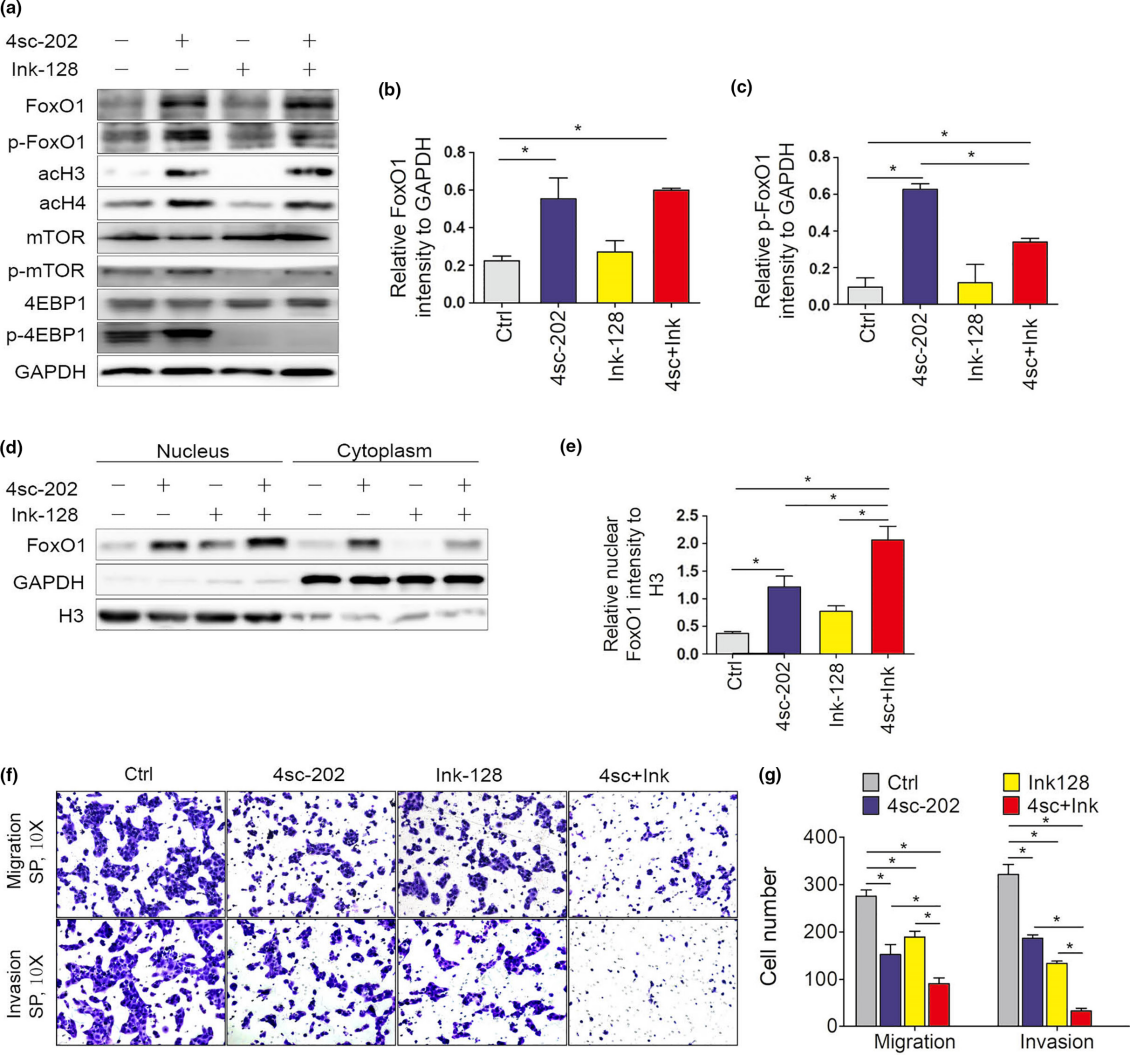
4sc‐202 synergized with Ink‐128 to activate FoxO1 and suppress tumor cell survival. (a) HSC6 cells treated with 4sc‐202, Ink‐128, or a combination of both for 24 hr. Cell proteins were analyzed by western blotting, with GAPDH expression used as a loading control. (b, c) Relative amounts of total FoxO1 (b) and phosphorylated FoxO1 (p‐FoxO1) (c) were calculated by normalizing their expression levels to GAPDH and comparing the effects of each treatment with those in DMSO‐treated cells. (d) Cells treated with 4sc‐202, Ink‐128, or a combination of both for 24 hr. Nuclear and cytoplasmic fractions were analyzed by western blotting. GAPDH and nuclear H3 were detected as loading controls for the cytoplasmic and nuclear fractions, respectively. (e) Relative amounts of nuclear FoxO1 were calculated by normalizing to H3 expression levels and comparing the normalized FoxO1 levels after each treatment with the levels with those in DMSO‐treated cells. (f, g) HSC6 cells treated with 4sc‐202, Ink‐128, or a combination of both for 24 hr. The migration and invasion abilities were examined by performing transwell assays. Representative images of migration or invasion to the lower surface (f) and the mean numbers of migrated or invading cells (g) are shown

In addition, the observation that both 4sc‐202 and Ink‐128 promoted FoxO1 nuclear translocation through different mechanisms increases the likelihood that they might synergistically suppress OSCC metastasis. We evaluated the effects of combined 4sc‐202 and Ink‐128 treatment on FoxO1 expression and phosphorylation in OSCC cells to test this possibility. Treatment with 4sc‐202 increased the protein levels of FoxO1 and acetylated histones (H3 and H4) (Figure 4a,b). Following induction by 4sc‐202, the FoxO1 protein showed a high degree of phosphorylation. The mTOR inhibitor, Ink‐128, did not increase the absolute expression level of FoxO1, but it accelerated FoxO1 accumulation in the nucleus, which was enhanced by 4sc‐202 (Figure 4a–c). Ink‐128 also suppressed the phosphorylation of mTOR and 4EBP1, which are known target genes of the mTOR signaling pathway. Therefore, 4sc‐202 and Ink‐128 cooperatively triggered FoxO1 dephosphorylation in OSCC cells.

Because FoxO1 dephosphorylation increased its nuclear localization, we tested whether the combined effect of 4sc‐202 with Ink‐128 could promote FoxO1 nuclear translocation. HSC6 were treated with 4sc‐202, Ink‐128, or a combination of both drugs for 24 hr, and then, the respective cytoplasmic and nuclear levels of FoxO1 were measured. As observed by western blotting (Figure 4d,e), combined treatment with 4sc‐202 and Ink‐128 led to increased FoxO1 nuclear accumulation, when compared with control cells or cells treated with either drug alone. These data suggest that 4sc‐202 and Ink‐128 synergistically led to increased FoxO1 protein dephosphorylation and promoted FoxO1 activation.

The synergistic effect of 4sc‐202 and Ink‐128 observed at the biochemical level suggests that 4sc‐202 and Ink‐128 might synergistically inhibit OSCC EMT. To test this possibility, we treated HSC6 with 4sc‐202, Ink‐128, or a combination of both drugs. Both drugs cooperated to reduce invasion and migration more than treatment with either 4sc‐202 or Ink‐128 alone (Figure 4f,g). These data suggest that 4sc‐202 and Ink‐128 synergistically inhibited OSCC cell metastasis.

### 3.4 FoxO1 inhibited EMT by negatively regulating the transcription factor, Twist family bHLH transcription factor 1 (Twist1)

**FIGURE 5:**
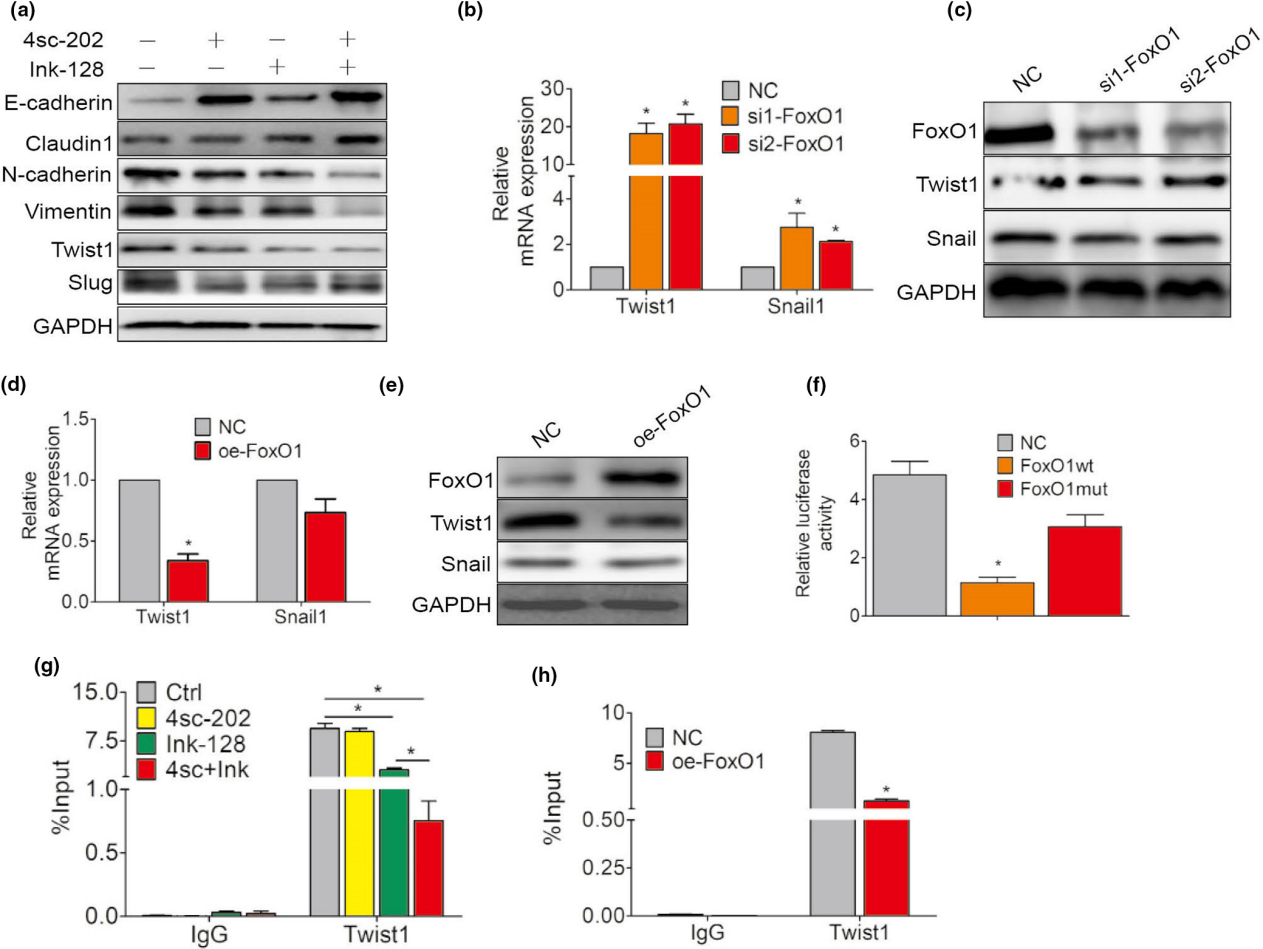
FoxO1 inhibited EMT by negatively regulating the EMT‐induced transcription factor Twist1. (a) Expression of the EMT‐associated proteins E‐cadherin, claudin‐1, N‐cadherin, vimentin, Twist1, and Snail was examined by western blotting after HSC6 cells were treated with 4sc‐202 or/and Ink‐128 for 24 hr. B, C HSC6 cells transfected with si‐NC or si‐FoxO1. (b) Twist1 and Snail mRNA levels measured by qRT‐PCR. (c) Expression levels of FoxO1, Twist1, and Snail detected by western blotting. GAPDH expression was detected as an internal control. (d, e) HSC6 cells transfected with an empty plasmid or a vector encoding oe‐FoxO1. (d) Twist1 and Snail mRNA levels measured by qRT‐PCR. (e) Expression levels of FoxO1, Twist1, and Snail were detected by western blotting. GAPDH expression was detected as an internal control. (f) The effects of Twist1 mimics in HSC6 cells on the luciferase activities of a reporter construct encoding the wild‐type 3′‐untranslated region (UTR) sequence or a mutant 3′‐UTR sequence. G ChIP‐qPCR analysis identified FoxO1 at the Twist1 gene locus after 4sc‐202 or/and Ink‐128 for 24 hr treatment. H ChIP‐qPCR analysis identified FoxO1 at the Twist1 gene locus after FoxO1‐overexpressing

FoxO1 proteins orchestrate the activities of EMT‐TFs, thereby modulating the EMT process. Snail, Snail, ZEB1, ZEB2, and Twist1 are key regulators of the EMT program. We searched for FoxO1‐regulated genes using transcription factor‐prediction programs, including hTHtarget (http://bioinfo.life.hust.edu.cn/hTFtarget/) and JASPAR (http://jaspar.genereg.net/). We identified two transcription factors, Snail and Twist1, as potential key mediators of FoxO1‐induced mRNA level changes. Following combined treatment with Ink‐128 and 4sc‐202, the Twist1 protein level decreased, when compared with cells treated with control cells or cells treated with either drug alone. However, 4sc‐202 had no effect on the expression of Snail (Figure 5a and Figure S2a).

We examined the expression levels of these transcription factors and their potential interactions with FoxO1 during EMT. Knocking down FoxO1 expression resulted in a moderate increase in Twist1 mRNA levels (Figure 5b,c; Figure S2b), whereas FoxO1 overexpression resulted in decreased Twist1 mRNA expression (Figure 5d,e; Figure S2c) compared with control. Snail expression was not noticeably affected (Figure 5b–e). These findings suggest that FoxO1 might reverse EMT by interacting with Twist1 in HSC6 cells. The activity of the Twist1 promoter was then assessed, using reporter constructs. We compared the effects of a DNA binding‐defective mutant of FoxO1 and overexpression of wild‐type FoxO1 on Twist1 promoter activity. As shown in Figure 5f, FoxO1 overexpression reduced luciferase reporter activities. However, mutations in the FoxO1‐binding sites completely restored the luciferase activity, suggesting that FoxO1 suppressed Twist1 transcription by directly binding to the Twist1 promoter. In agreement with our reporter construct results, chromatin immunoprecipitation‐quantitative polymerase chain reaction (ChIP‐qPCR) analysis revealed that the binding activity of FoxO1 at the Twist1 gene locus decreased upon combined treatment with the 4sc‐202 and ink‐128 (Figure 5g). Similarly, FoxO1‐expressing HSC6 cells showed an 84.4% decrease in Twist1 promoter activity compared with control cells (Figure 5h). In summary, the results clearly indicated that FoxO1 was crucial for inhibiting Twist1 promoter activity.

## 4 DISCUSSION

Cancer metastasis and relapse are responsible for the poor prognoses of patients with OSCC (Smith et al., 2013) and represent significant challenges in cancer management. A promising new therapeutic strategy is urgently needed. In this study, we developed a novel strategy for reversing EMT and treatment resistance by targeting FoxO1 in OSCC.

We found that 4sc‐202 exhibited antitumor activity by inhibiting proliferation and promoting apoptosis and could block invasion and migration of OSCC cell lines (Pinkerneil et al., 2016). RNA expression analysis revealed that 4sc‐202 markedly reduce FoxO1‐targeted genes in OSCC cells. Furthermore, 4sc‐202 significantly increased the FoxO1 mRNA and protein levels. There are several mechanisms whereby 4sc‐202 might induce FoxO1. As mentioned above, FoxO1 mRNA expression decreased during OSCC formation (Zheng et al., 2019), suggesting that high FoxO1 levels may be adverse for tumor growth. FoxO1 might restore E‐cadherin expression and reverse EMT by inhibiting the interaction between miR‐9 and E‐cadherin mRNA (Ma et al., 2018) or by suppressing the TGF‐β1–Smad3–ILK pathway (Du et al., 2016).

FoxO1 serves a necessary function in HDAC inhibitor‐based suppression of larynx carcinoma invasion and metastasis by blocking MMP7 (Ding et al., 2014) or by binding the ZEB2 promoter and reversing ZEB2‐induced EMT (Dong et al., 2017). However, FoxO1 activated by ERK2 can promote the migratory and invasive potential of tumor cells by inducing EMT, but can also inhibit tumor proliferation (Shin et al., 2019). In addition, FoxO1 is phosphorylated via the AKT‐mTOR pathway, which accelerates its nuclear translocation from the cytoplasm and results in decreased FoxO1 transcriptional activity (Ma et al., 2018). Our findings that FoxO1 upregulation was necessary for 4sc‐202‐dependent suppression of OSCC invasion and metastasis, and that FoxO1 downregulation lowered the responses to 4sc‐202, suggest that the FoxO1 pathway plays an important role in 4sc‐202‐mediated OSCC inhibition. It should be noted that 4sc‐202 can activate multiple pathways in OSCC simultaneously; thus, other proteins besides FoxO1 may participate in 4sc‐202‐mediated growth inhibition.

Data from many ongoing clinical trials suggest that HDAC inhibitors comprise a promising group of targeted anti‐cancer agents (D’Souza & Saranath, 2015). However, the benefits of 4sc‐202 alone are limited in many cancers (Fu et al., 2016; Pinkerneil et al., 2016; Zhijun et al., 2016), and the full potential of 4sc‐202 for treating cancer is probably better realized in combination with other molecules (Liang et al., 2019). 4sc‐202 can induce FoxO1 expression, and Akt–mTOR pathway activation can promote FoxO1 phosphorylation and prevent it from entering the nucleus (Ma et al., 2018). Data from our previous studies showed that inhibitors of the Akt–mTOR pathway also restrain OSCC EMT (Wang et al., 2017). We hypothesize that co‐treatment with mTOR antagonists and 4sc‐202 might synergistically reverse OSCC EMT.

Our results suggest that combined treatment with 4sc‐202 and ink‐128 synergistically activated FoxO1 and reversed OSCC EMT in vitro. The fact that both types of agents can effectively upregulate FoxO1 activity in patients with OSCC (4sc‐202 increased expression FoxO1; Ink‐128 promoted nuclear translocation of FoxO1) suggests that FoxO1 is crucial for sensitizing OSCC cells to combination treatment with 4sc‐202 and Ink‐128. Furthermore, the fact that the agents modulate FoxO1 activity through different molecular mechanisms increases the likelihood that these agents could cooperate to trigger robust tumor regression.

Cancer metastasis is a key factor leading to poor prognosis (Ma et al., 2016). The fact that EMT is a vital step for promoting cancer metastasis has been widely documented. FoxO1 can coordinate EMT‐related pathways and EMT‐TFs, thereby regulating the EMT process (He et al., 2017). The mechanisms underlying the FoxO1‐associated EMT process are complex and little is known regarding these mechanisms in OSCC. To our knowledge, this is the first report to show that the transcription factor Twist1 plays a role in FoxO1‐mediated metastasis inhibition, and our findings provide new clues for understanding EMT regulation. The results of this study are consistent with previous reports demonstrating that FoxO1 inhibited Twist1 mRNA expression in cancer cells (Jie et al., 2020). In addition, Twist1 was previously found to be highly expressed in lung metastatic nodules originating from OSCC cells (Yochum et al., 2019), and Twist1 overexpression is related to OSCC recurrence (Wei et al., 2015). Twist1 can bind directly to the E‐cadherin promoter and strongly inhibit E‐cadherin expression, indicating that Twist1 promotes tumor cell invasion and metastasis (Fukusumi et al., 2018). In the present work, we demonstrated that FoxO1 and Twist1 interacted with each other. Our data from ChIP experiments suggest that FoxO1 inhibited the invasion and metastasis of tumor cells by transcriptionally regulating the Twist1 promoter. This is the first report to demonstrate a relationship between FoxO1 and Twist1.

In summary, our findings provide evidence that combination treatment with the HDAC inhibitor 4sc‐202 and the mTOR inhibitor Ink‐128 effectively reduced EMT by simultaneous inducing FoxO1 and repressing EMT inducers (Twist1) in OSCC cells. These findings suggest this drug combination as a promising new therapeutic strategy for OSCC, although further clinical investigation is necessary to verify this hypothesis.

## CONFLICT OF INTEREST

None to declare.

## AUTHOR CONTRIBUTIONS

**Xi Yang:** Conceptualization; Investigation; Methodology; Writing‐original draft. **Tianyu Sun:** Conceptualization; Investigation; Methodology; Writing‐original draft. **Yajing Zhao:** Software. **Shuying Liu:** Software. **Xueyi Liang:** Conceptualization; Funding acquisition; Project administration; Supervision; Writing‐review & editing.

### PEER REVIEW

The peer review history for this article is available at https://publons.com/publon/10.1111/odi.13860.

## Supporting information

Fig S1

Fig S2

Table S1‐S3

File S1

## References

[odi13860-bib-0001] Ali, J. , Sabiha, B. , Jan, H. U. , Haider, S. A. , Khan, A. A. , & Ali, S. S. (2017). Genetic etiology of oral cancer. Oral Oncology, 70, 23–28. 28622887 10.1016/j.oraloncology.2017.05.004

[odi13860-bib-0002] Castilho, R. M. , Squarize, C. H. , & Almeida, L. O. (2017). Epigenetic modifications and head and neck cancer: Implications for tumor progression and resistance to therapy. International Journal of Molecular Sciences, 18, 1506. 28704968 10.3390/ijms18071506 PMC5535996

[odi13860-bib-0003] Chang, H. , Chiang, C. , Hung, H. , Lin, C. , Deng, Y. , & Kuo, M. Y. (2009). Histone deacetylase 2 expression predicts poorer prognosis in oral cancer patients. Oral Oncology, 45, 610–614. 18951835 10.1016/j.oraloncology.2008.08.011

[odi13860-bib-0004] D’Souza, W. , & Saranath, D. (2015). Clinical implications of epigenetic regulation in oral cancer. Oral Oncology, 51(12), 1061–1068. 10.1016/j.oraloncology.2015.09.006 26421863

[odi13860-bib-0005] Ding, H. , Zhu, Y. , Chu, T. , & Wang, S. (2014). Epidermal growth factor induces FoxO1 nuclear exclusion to activate MMP7‐mediated metastasis of larynx carcinoma. Tumour Biology, 35, 9987–9992. 25008564 10.1007/s13277-014-2067-x

[odi13860-bib-0006] Dong, T. , Zhang, Y. , Chen, Y. , Liu, P. , An, T. , Zhang, J. , Yang, H. , Zhu, W. , & Yang, X. (2017). FOXO1 inhibits the invasion and metastasis of hepatocellular carcinoma by reversing ZEB2‐induced epithelial‐mesenchymal transition. Oncotarget, 8, 1703. 27924058 10.18632/oncotarget.13786 PMC5352090

[odi13860-bib-0007] Du, M. , Wang, Q. , Li, W. , Ma, X. , Wu, L. , Guo, F. , Zhao, S. , Huang, F. , Wang, H. , & Qin, G. (2016). Overexpression of FOXO1 ameliorates the podocyte epithelial‐mesenchymal transition induced by high glucose in vitro and in vivo. Biochemical and Biophysical Research Communications, 471, 416–422. 26902117 10.1016/j.bbrc.2016.02.066

[odi13860-bib-0008] Fu, M. , Wan, F. , Li, Z. , & Zhang, F. (2016). 4SC‐202 activates ASK1‐dependent mitochondrial apoptosis pathway to inhibit hepatocellular carcinoma cells. Biochemical and Biophysical Research Communications, 471, 267–273. 26773495 10.1016/j.bbrc.2016.01.030

[odi13860-bib-0009] Fukusumi, T. , Guo, T. W. , Sakai, A. , Ando, M. , Ren, S. , Haft, S. , Liu, C. , Amornphimoltham, P. , Gutkind, J. S. , & Califano, J. A. (2018). The NOTCH4‐HEY1 pathway induces epithelial mesenchymal transition in head and neck squamous cell carcinoma. Clinical Cancer Research, 24, 619–633. 29146722 10.1158/1078-0432.CCR-17-1366 PMC6171749

[odi13860-bib-0010] He, Y. , Northey, J. J. , Pelletier, A. , Kos, Z. , Meunier, L. , Haibe‐Kains, B. , Mes‐Masson, A. M. , Cote, J. F. , Siegel, P. M. , & Lamarche‐Vane, N. (2017). The Cdc42/Rac1 regulator CdGAP is a novel E‐cadherin transcriptional co‐repressor with Zeb2 in breast cancer. Oncogene, 36, 3490–3503. 28135249 10.1038/onc.2016.492 PMC5423781

[odi13860-bib-0011] Jie, M. , Wu, Y. , Gao, M. , Li, X. , Liu, C. , Ouyang, Q. , Tang, Q. , Shan, C. , Lv, Y. , Zhang, K. , Dai, Q. , Chen, Y. , Zeng, S. , Li, C. , Wang, L. , He, F. , Hu, C. , & Yang, S. (2020). CircMRPS35 suppresses gastric cancer progression via recruiting KAT7 to govern histone modification. Molecular Cancer, 19. 10.1186/s12943-020-01160-2 PMC7066857 32164722

[odi13860-bib-0012] Kurth, I. , Hein, L. , Mabert, K. , Peitzsch, C. , Koi, L. , Cojoc, M. , Kunz‐Schughart, L. , Baumann, M. , & Dubrovska, A. (2015). Cancer stem cell related markers of radioresistance in head and neck squamous cell carcinoma. Oncotarget, 6, 34494–34509. 26460734 10.18632/oncotarget.5417 PMC4741468

[odi13860-bib-0013] Liang, X. , Deng, M. , Zhang, C. , Ping, F. , Wang, H. , Wang, Y. , Fan, Z. , Ren, X. , Tao, X. , Wu, T. , Xu, J. , Cheng, B. , & Xia, J. (2019). Combined class I histone deacetylase and mTORC1/C2 inhibition suppresses the initiation and recurrence of oral squamous cell carcinomas by repressing SOX2. Cancer Letters, 454, 108–119. 30981761 10.1016/j.canlet.2019.04.010

[odi13860-bib-0014] Ma, Z. , Xin, Z. , Hu, W. , Jiang, S. , Yang, Z. , Yang, Y. , Yan, X. , Li, X. , & Chen, F. (2018). Forkhead box O proteins: Crucial regulators of cancer EMT. Seminars in Cancer Biology, 50, 21–31. 29427645 10.1016/j.semcancer.2018.02.004

[odi13860-bib-0015] Ma, Z. , Yang, Y. , Fan, C. , Han, J. , Wang, D. , Di, S. , Hu, W. , Liu, D. , Li, X. , Reiter, R. J. , & Yan, X. (2016). Melatonin as a potential anticarcinogen for non‐small‐cell lung cancer. Oncotarget, 7, 46768–46784. 27102150 10.18632/oncotarget.8776 PMC5216835

[odi13860-bib-0016] Malone, C. F. , Emerson, C. , Ingraham, R. , Barbosa, W. , Guerra, S. , Yoon, H. , Liu, L. L. , Michor, F. , Haigis, M. , Macleod, K. F. , Maertens, O. , & Cichowski, K. (2017). mTOR and HDAC inhibitors converge on the TXNIP/Thioredoxin pathway to cause catastrophic oxidative stress and regression of RAS‐driven tumors. Cancer Discovery, 7, 1450–1463. 28963352 10.1158/2159-8290.CD-17-0177 PMC5718976

[odi13860-bib-0017] Pastushenko, I. , Brisebarre, A. , Sifrim, A. , Fioramonti, M. , Revenco, T. , Boumahdi, S. , Van Keymeulen, A. , Brown, D. , Moers, V. , Lemaire, S. , De Clercq, S. , Minguijón, E. , Balsat, C. , Sokolow, Y. , Dubois, C. , De Cock, F. , Scozzaro, S. , Sopena, F. , Lanas, A. , … Blanpain, C. (2018). Identification of the tumour transition states occurring during EMT. Nature, 556, 463–468. 29670281 10.1038/s41586-018-0040-3

[odi13860-bib-0018] Pinkerneil, M. , Hoffmann, M. J. , Kohlhof, H. , Schulz, W. A. , & Niegisch, G. (2016). Evaluation of the therapeutic potential of the novel isotype specific HDAC inhibitor 4SC‐202 in urothelial carcinoma cell lines. Target Oncology, 11, 783–798. 10.1007/s11523-016-0444-7 PMC5153417 27250763

[odi13860-bib-0019] Pradella, D. , Naro, C. , Sette, C. , & Ghigna, C. (2017). EMT and stemness: Flexible processes tuned by alternative splicing in development and cancer progression. Molecular Cancer, 16, 8. 10.1186/s12943-016-0579-2 28137272 PMC5282733

[odi13860-bib-0021] Shin, S. , Buel, G. R. , Nagiec, M. J. , Han, M. , Roux, P. P. , Blenis, J. , & Yoon, S. (2019). ERK2 regulates epithelial‐to‐mesenchymal plasticity through DOCK10‐dependent Rac1/FoxO1 activation. Proceedings of the National Academy of Sciences, 116, 2967–2976. 10.1073/pnas.1811923116 PMC6386703 30728292

[odi13860-bib-0022] Siegel, R. L. , Miller, K. D. , & Jemal, A. (2018). Cancer statistics. CA: A Cancer Journal for Clinicians, 68, 7–30. 29313949 10.3322/caac.21442

[odi13860-bib-0023] Smith, A. , Teknos, T. N. , & Pan, Q. (2013). Epithelial to mesenchymal transition in head and neck squamous cell carcinoma. Oral Oncology, 49, 287–292. 23182398 10.1016/j.oraloncology.2012.10.009 PMC3586749

[odi13860-bib-0024] Wang, H. , Liang, X. , Li, M. , Tao, X. , Tai, S. , Fan, Z. , Wang, Z. , Cheng, B. , & Xia, J. (2017). Chemokine (CC motif) ligand 18 upregulates Snail expression to promote stem‐cell like features by activating the mammalian target of rapamycin pathway in oral squamous cell carcinoma. Cancer Science, 108, 1584–1593. 28574664 10.1111/cas.13289 PMC5543498

[odi13860-bib-0025] Wei, S. C. , Fattet, L. , Tsai, J. H. , Guo, Y. , Pai, V. H. , Majeski, H. E. , Chen, A. C. , Sah, R. L. , Taylor, S. S. , Engler, A. J. , & Yang, J. (2015). Matrix stiffness drives epithelial–mesenchymal transition and tumour metastasis through a TWIST1–G3BP2 mechanotransduction pathway. Nature Cell Biology, 17, 678–688. 25893917 10.1038/ncb3157 PMC4452027

[odi13860-bib-0026] Yochum, Z. A. , Cades, J. , Wang, H. , Chatterjee, S. , Simons, B. W. , O Brien, J. P. , Khetarpal, S. K. , Lemtiri‐Chlieh, G. , Myers, K. V. , Huang, E. H. B. , Rudin, C. M. , Tran, P. T. , & Burns, T. F. (2019). Targeting the EMT transcription factor TWIST1 overcomes resistance to EGFR inhibitors in EGFR‐mutant non‐small‐cell lung cancer. Oncogene, 38, 656–670. 30171258 10.1038/s41388-018-0482-y PMC6358506

[odi13860-bib-0027] Zheng, M. , Cao, M. X. , Luo, X. J. , Li, L. , Wang, K. , Wang, S. S. , Wang, H. F. , Tang, Y. J. , Tang, Y. L. , & Liang, X. H. (2019). EZH2 promotes invasion and tumour glycolysis by regulating STAT3 and FoxO1 signalling in human OSCC cells. Journal of Cellular and Molecular Medicine, 23, 6942–6954. 31368152 10.1111/jcmm.14579 PMC6787444

[odi13860-bib-0028] Zhijun, H. , Shusheng, W. , Han, M. , Jianping, L. , Li‐Sen, Q. , & Dechun, L. (2016). Pre‐clinical characterization of 4SC‐202, a novel class I HDAC inhibitor, against colorectal cancer cells. Tumour Biology, 37, 10257–10267. 26831668 10.1007/s13277-016-4868-6

[odi13860-bib-0029] Zhuang, Z. , Xie, N. , Hu, J. , Yu, P. , Wang, C. , Hu, X. , Han, X. , Hou, J. , Huang, H. , & Liu, X. (2017). Interplay between DeltaNp63 and miR‐138‐5p regulates growth, metastasis and stemness of oral squamous cell carcinoma. Oncotarget, 8, 21954–21973. 28423539 10.18632/oncotarget.15752 PMC5400637

