# Supplementary figures and images for: 4sc‐202 and Ink‐128 cooperate to reverse the epithelial to mesenchymal transition in OSCC
Source: Oral Dis. 2021 May 4;28(8):2139–48. doi: 10.1111/odi.13860 (PMC10184781; doi:10.1111/odi.13860)

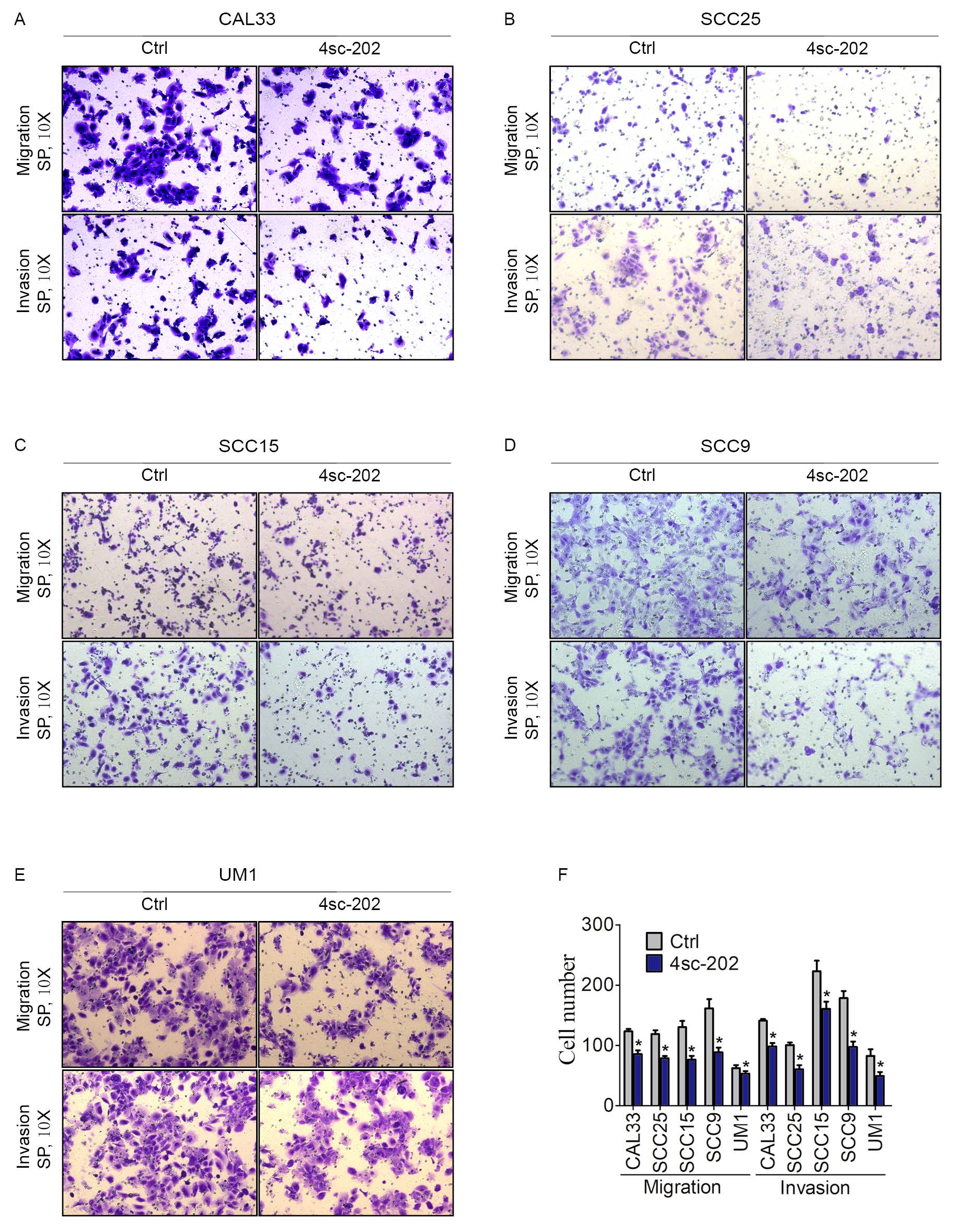

Supplement: Supplementary file 1 — Fig S1 [file ODI-28-2139-s002.tif]

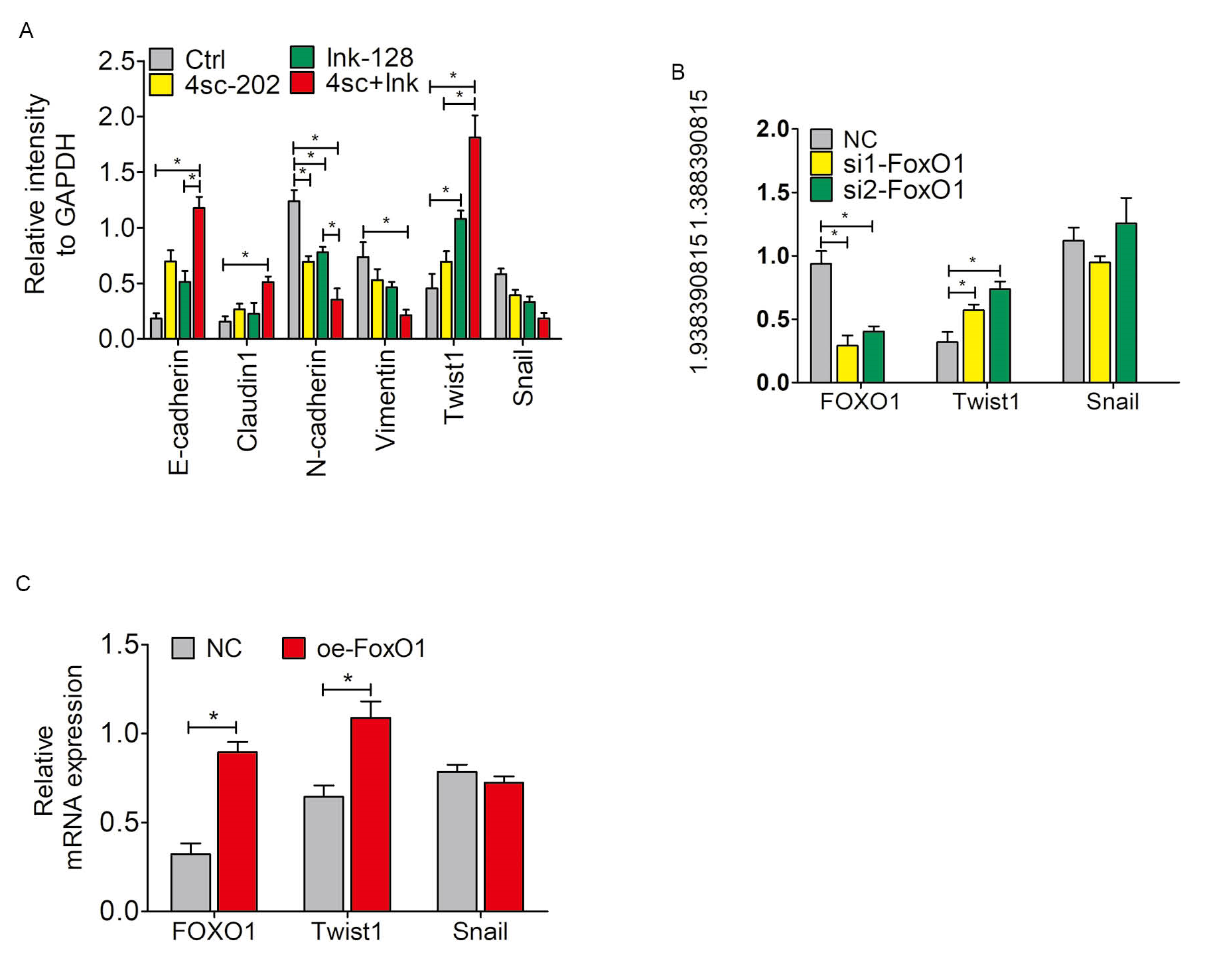

Supplement: Supplementary file 2 — Fig S2 [file ODI-28-2139-s001.tif]
