# Supplementary material for: 4sc‐202 and Ink‐128 cooperate to reverse the epithelial to mesenchymal transition in OSCC
Source: Oral Dis. 2021 May 4;28(8):2139–48. doi: 10.1111/odi.13860 (PMC10184781; doi:10.1111/odi.13860)
Supplement: Supplementary file 3 — Table S1‐S3 [file ODI-28-2139-s003.doc]

Supplementary Table 1. Antibodies used in the current study

| **Antibody** | **Company** | **Product number** |
| --- | --- | --- |
| E-Cadherin | Cell Signaling | 3195 |
| Claudin-1 | Cell Signaling | 13255 |
| N-Cadherin | Cell Signaling | 13116 |
| Vimentin | Cell Signaling | 5741 |
| Twist1 | Cell Signaling | 46702 |
| Twist1 (used for immunohistochemistry) | Santa Cruz | sc- 81417 |
| FoxO1 | Cell Signaling | 2880 |
| Snail | Cell Signaling | 3879 |
| acetyl histone H3 | Millpore | 06-599 |
| acetyl histone H4 | Millpore | 06-866 |
| GAPDH | Cell Signaling | 2118L |
| α-Tubulin | affinity | AF0524 |
| Anti-rabbit IgG, HRP-linked Antibody | Cell Signaling | 7074 |
| Anti-mouse IgG, HRP-linked Antibody | Cell Signaling | 7076 |

Supplementary Table 2. Primers used for SYBR Green-based qRT-PCR analysis

| **Gene** | **primer** | **Primer Sequence (5’🡪 3’)** |
| --- | --- | --- |
| Homo-FoxO1 | Forward | GGTTAGTGAGCAGGTTAC |
|  | Reverse | AAAGGGAGTTGGTGAAAG |
| Homo-Twist1 | Forward | GCTTGAGGGTCTGAATCTTGCT |
|  | Reverse | GTCCGCAGTCTT ACGAGGAG |
| Homo-Snail | Forward | TTCAACTGCAAATACTGCAACAAG |
|  | Reverse | CAGTGTGGGTCCGGACATG |
| Homo-GAPDH | Forward | GCACCGTCAAGGCTGACAAC |
|  | Reverse | TGGTGAAGACGCCAGTGGA |

**Supplementary Table 3. ChIP primers used for SYBR Green-based qRT-PCR analysis**

| **Gene** | **primer** | **Primer Sequence (5’🡪 3’)** |
| --- | --- | --- |
| Homo-Twist1 | Forward | CACATTCAACAGGCAGCAG |
|  | Reverse | CGCGAGCCCAGAACTTAAC |
| Homo-GAPDH | Forward | GGCTCCCACCTTTCTCATCC |
|  | Reverse | GGCCATCCACAGTCTGG |
