# Supplementary material for: 4sc‐202 and Ink‐128 cooperate to reverse the epithelial to mesenchymal transition in OSCC
Source: Oral Dis. 2021 May 4;28(8):2139–48. doi: 10.1111/odi.13860 (PMC10184781; doi:10.1111/odi.13860)
Supplement: Supplementary file 4 — File S1 [file ODI-28-2139-s004.docx]

**Supporting files**

**Fig.S1** 4sc-202 Inhibit invasion and migration in OSCC in Vitro. **A-E** CAL33, SCC25,SCC15,SCC9 and UM1 cells were cultured in the absence or presence of 4sc-202 with the indicated concentrations of 4sc-202(1μm) for 24h, and then the migration and invasion abilities were examined by transwell assay. Representative migrated or invaded pictures to the lower surface **(A-E**) and the mean number of migrated or invaded cells were shown (**F**).

**Fig.S2** FoxO1 inhibited EMT by negatively regulating the EMT-induced transcription factor Twist1. **A** The relative expression intensity compared to the GAPDH are shown. **B** The relative expression intensity compared to the GAPDH internal control are shown. **C** Relative amounts of total FoxO1、Twist1、Snail were calculated by normalizing their expression levels to GAPDH and comparing the effects of each treatment with those cells transfected with an empty plasmid.
